# RIS-assisted near-field localization using practical phase shift model

**DOI:** 10.1038/s41598-024-54859-3

**Published:** 2024-02-22

**Authors:** Saber Hassouna, Muhammad Ali Jamshed, Masood Ur-Rehman, Muhammad Ali Imran, Qammer H. Abbasi

**Affiliations:** James Watt School of Engineering, University of Glasgow, Glasgow, UK

**Keywords:** Energy science and technology, Engineering, Energy science and technology, Engineering

## Abstract

Our research focuses on examining the problem of localizing user equipment (UE) in the uplink scenario using reconfigurable intelligent surfaces (RIS) based lens. We carry out a thorough analysis of the Fisher information matrix (FIM) and assess the influence of various RIS-based lens configurations using an actual RIS phase-dependent amplitude variations model. Furthermore, to reduce the complexity of the maximum likelihood (ML) estimator, a simple localization algorithm-based angular expansion is presented. Simulation results show superior localization performance when prior location information is available for directional and positional channel configurations. The position error bound (PEB) and the root mean square error (RMSE) are studied to evaluate the localization accuracy of the user utilizing the realistic RIS phase-dependent amplitude model in the near-field region. Furthermore, the achievable data rate is obtained in the same region using the realistic RIS phase-dependent amplitude model. It is noticed that adopting the actual RIS phase-dependent amplitude model under the near-field channel increases the localization error and degrades the data rate performance for amplitude value less than one so, the unity assumption of the RIS phase shift model used widely in the literature is inaccurate.

## Introduction

In the realm of wireless communication networks, radio localization presents a feasible substitute for acquiring user location data within environments where global positioning system (GPS) signals are unavailable^1^. With each successive generation of mobile communication, novel features are introduced to facilitate high-speed communication, while simultaneously enhancing the precision of localization capabilities^2,3^. Radio localization techniques operate under the fundamental concept that the radio signals contain valuable information regarding the positional data of network nodes. In fourth generation (4G) systems, users make use of time-of-arrival (ToA) estimation^4^ in relation to each base station (BS). This estimation relies on factors such as the distance between the user and the BS, as well as the clock bias at the user. Using ToA from at least four line-of-sight (LoS) BSs, users can calculate three-time difference of arrival (TDoA) measurements to determine their three-dimensional (3D) location. In fifth generation (5G) systems that operate in millimeter wave (mm-Wave) frequency bands, both the BS and potentially the user are equipped with multiple antennas. In this scenario, the channel is characterized by both delays and angles. The receiver determines the angle-of-arrival (AoA), while the transmitter determines the angle-of-departure (AoD). This parameterization of the channel considers both the spatial angle and the delay in the propagation of signals^5–7^.

The growing prevalence of applications such as smart factories, automated/assisted driving, and augmented reality has led to increasingly stringent requirements for positioning accuracy in 5G and sixth generation (6G) communication networks. In 5G, the wider bandwidth and larger antenna arrays have improved localization accuracy, making it possible to efficiently localize devices using just one BS^8,9^. Moreover, the reliability of localization provided by 5G and 6G communications is of utmost significance. As 5G and 6G systems can operate in high-frequency mm-Wave and THz bands, the links between devices are susceptible to obstacles. Since LoS propagation is typically necessary for precise location estimation, existing localization methods yield significant estimation errors if the LoS link is obstructed^10^.

Alongside their advantages for communication purposes, reflecting intelligent surfaces (RIS)s offer reliable and highly precise position estimation capabilities with low cost and high energy efficiency^11–13^. When the LoS link is obstructed, an RIS can establish a virtual LoS link, allowing for accurate delay measurements when utilizing wideband signals^14^. Unlike non-reconfigurable scatterers present in the environment, RISs have the ability to adjust the phase shifts of their reflecting elements, resulting in a significant beamforming gain. Additionally, RISs offer a large number of elements, further contributing to the high resolution achieved in the localization process. This capability enables RISs to provide enhanced resolution in AoA for uplink localization or AoD for downlink localization^15^.

For communication and localization applications involving RISs, it is crucial to have precise and well-defined control over the RIS. This requires the development of proper and straightforward models for RIS phase control. These models should ideally incorporate various factors such as the effects of mutual coupling^16,17^, calibration, quantization^18^ and power losses per element^19^. Most existing studies on RIS localization have focused on ideal phase shifters and have neglected the impairments mentioned above. As a result, it remains unknown how these proposed localization approaches would perform when these impairments are taken into account. However, understanding the impact of these impairments is essential as it can significantly influence the effectiveness and reliability of RIS-based localization methods^20,21^. The current state-of-the-art to intelligent surfaces-based localization involves investigations employing RIS in either receive mode^11^ or reflection mode^22,23^. In receive mode, a sizable intelligent surface is utilized to determine the location of a user positioned in front of it, applicable in both near-field and far-field scenarios^11,11^. On the other hand, when operating in reflection mode, a strategy is employed to modify the RIS reflection coefficient. This alteration enhances the received signal strength (RSS) at various points, thereby improving localization accuracy^24^. In contrast, a different approach is presented in^22^, where the authors utilize an RIS to support positioning and communication in mm-Wave frequency bands, assuming that the mobile device is in the far-field with respect to the RIS. However, this assumption may not always hold true, especially when dealing with large surfaces and arrays relative to the distance. Consequently, the models involved become less accurate, as the mobile device is situated in the Fresnel region rather than the Fraunhofer region. In the Fresnel region, the wavefront exhibits significant curvature and cannot be approximated as a plane wave. Additionally, disregarding the spherical wavefront omits crucial information regarding the mobile device’s location and orientation^25–27^. Under the spherical wavefront channel model, only a limited number of studies have explored the RIS-aided radio localization problem. One notable contribution is the development of a near-field codebook in^28^ designed for extremely large-scale RIS beam training. This involves dividing the two-dimensional (2D) plane into several sampled points in the XY coordinate system. Furthermore, the authors of^29^ demonstrated that the characteristics of the transmitted signal, such as the transmit antenna type, size, and orientation, can significantly impact received signals in the near field. Considering these factors is essential for achieving high-precision localization.

In this paper we extended the work in^21^. Our research focuses on the exploration of 3D localization using a simplified RIS lens design, as described in reference^25^. This design incorporates adjustable RIS lenses and a sole antenna connected to a receiving radio frequency (RF) chain. We address the issue of RIS-aided geometric near-field localization in scenarios where LoS blockage is present. To tackle this challenge, we presented Fisher information analysis with a closed form expression of the Fisher information matrix (FIM), showing the dependence of the position error bound (PEB) on the RIS phase profiles. We used three RIS phase profiles random, directional and positional configurations to demonstrate the role of RIS in localization and communication in the near-field regime. The RIS phase profiles are designed taking into consideration the amplitude and phase responses of the RIS by adopting the practical phase-dependent amplitude model^30^. The random profile gives uniform signal-to-noise ratio (SNR) in the deployment area while the directional and positional increase the SNR towards the user location. Furthermore, we develop a simple localization scheme to reduce the complexity of the maximum likelihood (ML) estimator.The achievable data rate degrades with distance in the near-filed region and this coincides with localization error behavior which increases gradually with distance from the RIS. Both achievable data rate and localization error show inferior performance when adopting the RIS phase-dependent amplitude model for amplitude value less than one so, the unity assumption of the RIS phase shift model used widely in the literature leads to over-optimistic and incorrect localization and communication performance results.

**Figure 1:**
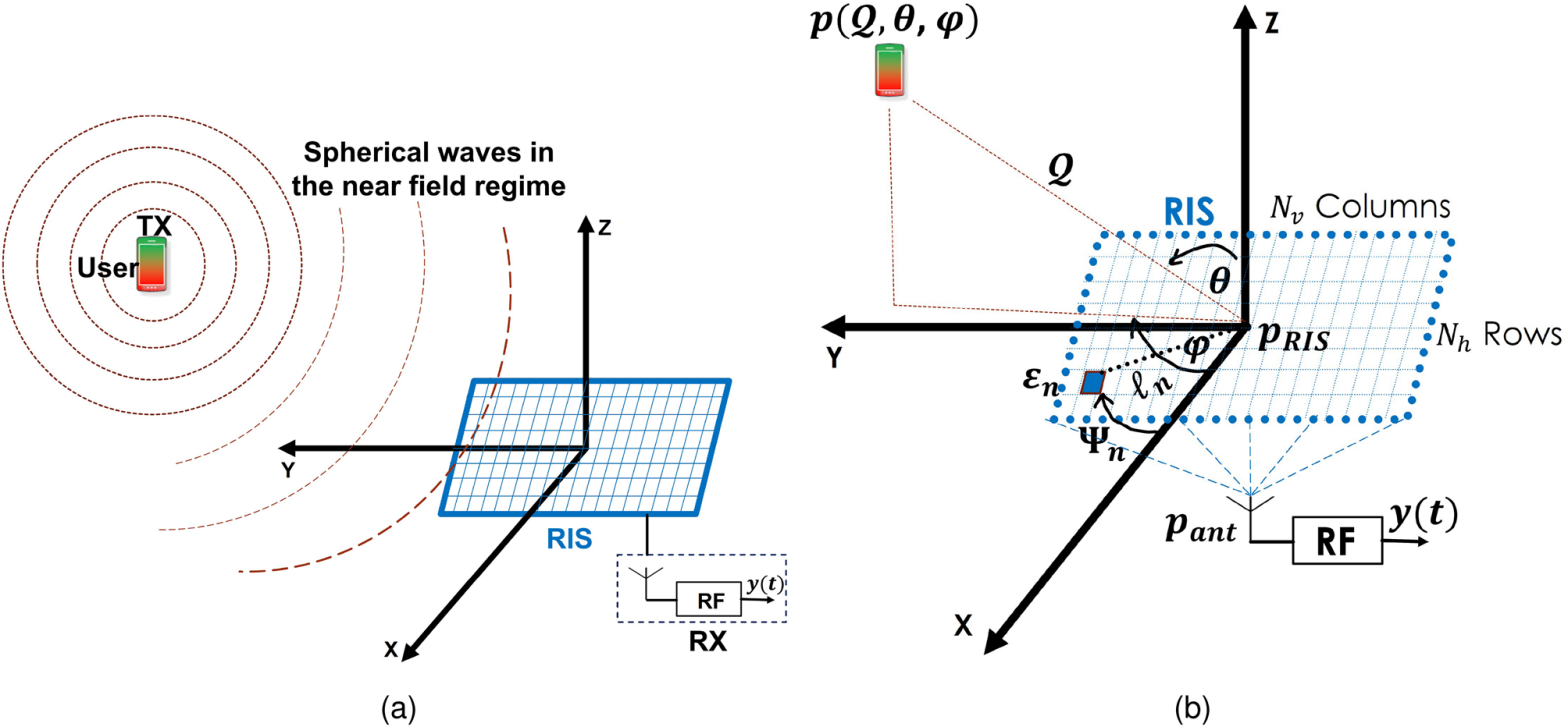
(**a**) System setup, (**b**) coordinate system.

The rest of the paper is organized as follows: “System model” presents the system model which includes the signal and the RIS models in addition to the problem statement. The FIM analysis is given in “Fisher information matrix (FIM) analysis”, while the location estimation is shown in “Low complexity estimation”. “Achievable data rate in the near field regime” discusses the achievable data rate in the near-field regime. The simulation results and the conclusion are given in “Simulation results” and “Conclusion”, respectively.

### Notations

Bold letters are used to represent vectors, while bold capital letters are used for matrices. To indicate the element in the $\mathcal {B}$th row and $\mathcal {C}$th column of matrix *A*, it is expressed as $[{A}]_{\mathcal {B}, \mathcal {C}}$. The notation ${A}_{\mathcal {B}: \mathcal {C}, \mathcal {K}: \mathcal {L}}$ refers to a matrix that consists of rows from $\mathcal {B}$ to $\mathcal {C}$ and columns from $\mathcal {K}$ to $\mathcal {L}$, taken from matrix *A*. The $\odot$ operator denotes the Hadamard product of vectors, $\mathbb {E}\{.\}$ represents the expectation operator, and $\dagger$ denotes the pseudo-inverse of a matrix. The ones and zeros column vectors represent $\textbf{1}_N$ and $\textbf{0}_N$ respectively with size *N*. Lastly, the *f*(*d*) represents the probability density function (PDF) of a random vector *d*.

## System model

### Geometry and signal model

We provide a detailed description of the system model utilized for localization in 3D with the help of the RIS lens. Additionally, we will introduce the near-field channel and the realistic amplitude-dependent phase model to evaluate the performance of the PEB, root mean square error (RMSE) and the achievable data rate.

We examine a wireless setup comprising of a single user transmitting from position ${p}=[x, y, z]^{\top }$, and an *N*-element RIS lens positioned in the XY plane with reference point $[0,0,0]^{\top }$. The RIS lens is placed near a single antenna equipped with a corresponding RF chain for reception. The antenna is located at ${p}_{\text{ ant } } \in \mathbb {R}^3$. In Fig. 1a, the system setup is depicted to present the components of the communication system, while Fig. 1b presents the 3D coordinate system that shows the positional and angular information of the user and RIS. The spacing between the horizontal and vertical elements is set to $\lambda / 2$ where, $\lambda$ is the wavelength of the carrier frequency. The $n$ ^th^ element of the RIS lens is located at $\mathcal {E}_n=\left[ x_n, y_n, 0\right] ^{\top }\in \mathbb {R}^3$ which is equivalent to the spherical coordinate $\ell _n\left[ \cos \Psi _n, \sin \Psi _n, 0\right] ^{\top } \in \mathbb {R}^3$. The $\ell _n$ refers to the element’s distance from the RIS origin to the element *n* and $\Psi _n$ is the $n^\text {th}$ element azimuth angle as illustrated in Fig. 1b. Each element exhibits a directivity pattern described by $\mathcal {D}(\theta , \varphi )=\cos ^2(\varphi ) \cos (\theta )$, where $\varphi \in [0,2 \pi ]$ represents the azimuth angle between the X-axis and the vertical projection of *p* on the XY plane and $\theta \in [0, \pi / 2]$ represents the elevation angle between the Z-axis and the user location *p*. From this point forward, we will refer to the AoAs as $\theta$ and $\varphi$. Using the aforementioned notations, we define the wave vector ${k}(\varphi , \theta )=\frac{2 \pi }{\lambda }[\cos (\theta ) \cos (\varphi ), \cos (\theta ) \sin (\varphi ), \sin (\theta )]^{\top }$. To express the unknown position vector of the user, *p*, we can utilize the wave vector. It can be represented as ${p}=-\lambda \mathcal {Q} {k}(\theta , \varphi ) / 2 \pi$, where $\mathcal {Q}$ is the Euclidean distance between *p* and the reference location of the RIS, denoted as $\mathcal {Q} \triangleq \Vert {p}\Vert$. Additionally, we model the prior knowledge of the user’s location as a Gaussian PDF *f*(*p*). This PDF is characterized by a mean, $\mu _p \in \mathbb {R}^3$, and a covariance matrix ${c}_p \in \mathbb {R}^{3 \times 3}$. In the given scenario, the user transmits a narrowband signal $\mathcal {S}_t$ over a series of *T* transmissions to the receive antenna through the RIS elements. The transmitted signal follows the requirements that the $\mathbb {E}\left\{ \left| \mathcal {S}_t\right| ^2\right\} =E_s$. In general, we presume that $\mathcal {S}_t=\sqrt{E_s}$ for any given transmission *t*. The received signal at the output of the RF receiver can be represented mathematically for each time instance *t* as:

1 $$\begin{aligned} r_t=e^{j \vartheta } {h}_{\rm{ant}}^{\top } \textbf{v}_t({\Gamma }({p}) \odot {\mathcal {V}}({p})) \mathcal {S}_t+\mathcal {Z}_t, \end{aligned}$$

where, $\textbf{v}_t={\text {diag}}\left( v_{t, 0} \ldots v_{t, N-1}\right)$ is the reflection coefficients for *N* RIS lens elements at time *t* and $v_{t, n}$ represents the amplitude $\in (0,1]$ and phase shift $\in [0,2 \pi )$ for RIS element *n* at time *t*. The practical RIS phase shift model is provided next. Furthermore, $\vartheta =-\frac{2 \pi \mathcal {Q}}{\lambda }+\vartheta _{\rm{sync}}$ represents the phase synchronization between the receiver and transmitter. The vector ${h}_{\rm{ant}} \in \mathbb {C}^{N \times 1}$ comprises the gains of the propagation channels from the RIS to the receiver. The vector ${\Gamma }({p}) \ge \textbf{0}_N$ represents the amplitudes of the propagation channels between the RIS elements and the transmitter while, the channel phases can be represented by the vector ${\mathcal {V}}({p}) \in \mathbb {C}^{N \times 1}$. The noise vector $\mathcal {Z}_t$ represents uncorrelated zero-mean additive Gaussian noise with a variance of $N_0 / 2$ per real dimension. By introducing ${u}_t=\textbf{v}_t {h}_{\rm{ant}}$, we can define ${U}=\left[ {u}_1, {u}_2, \ldots , {u}_T\right] \in \mathbb {C}^{N \times T}$. Additionally, ${\mathcal {S}}=\left[ \mathcal {S}_1, \mathcal {S}_2, \ldots , \mathcal {S}_T\right] ^{\top }$ and ${\mathcal {Z}}=\left[ \mathcal {Z}_1, \mathcal {Z}_2, \ldots , \mathcal {Z}_T\right] ^{\top }$. In this context, the measurement vector ${r}=\left[ r_1, r_2, \ldots , r_T\right] ^{\top }$ can be represented as:

2 $$\begin{aligned} {r}=e^{j \vartheta } {\text {diag}}({\mathcal {S}}) {U}^{\top }({\Gamma }({p}) \odot {\mathcal {V}}({p}))+{\mathcal {Z}}. \end{aligned}$$

The model employed in signal processing literature for the localization of objects in the near-field is essentially the far-field model of electromagnetics, or a closely related approximation^29^. This work neglects the consideration of electromagnetic near-field effects within the Fraunhofer distance. In the near-field channel model, the steering vector ${\mathcal {V}}({p})$ for a specific position *p* is defined as:

3 $$\begin{aligned}{}[{\mathcal {V}}({p})]_n=\exp \left( -j \frac{2 \pi }{\lambda }\left( \left\| {p}-\mathcal {{E}}_n\right\| -\mathcal {Q}\right) \right) , \end{aligned}$$

and the amplitude ${\Gamma }({p})=\Gamma \textbf{1}_N$ is constant for all RIS elements. In the improved near-field channel model, however, the upper bound of the amplitudes ${\Gamma }({p})$ can be presented as per^26^:

4 $$\begin{aligned} {[}{\Gamma }({p})]_n^2=(4 \pi )^{-1} \sum _{\begin{array}{c} \textrm{x} \in \mathcal {X}_n \\ \textrm{y} \in \mathcal {Y}_n \end{array}} \frac{\textrm{xy}}{\left( \textrm{y}^2+z^2\right) B(\textrm{x}, \textrm{y})}+2 \tan ^{-1}\left( \frac{\textrm{xy}}{z^2 B(\textrm{x}, \textrm{y})}\right) , \end{aligned}$$

where, the terms, $\mathcal {X}_n=\left\{ \Delta / 2+\left( x_n-x\right) , \Delta / 2-\left( x_n-x\right) \right\}$, $\mathcal {Y}_n=\left\{ \Delta / 2+\left( y_n-y\right) , \Delta / 2-\left( y_n-y\right) \right\}$ and $B(\textrm{x}, \textrm{y})=\sqrt{x^2 / z^2+y^2 / z^2+1}$. Furthermore, $\Delta =\lambda / 2$ is the spacing between RIS elements so each $n$ ^th^ element of the RIS lens, with $n=1,2, \ldots , N$, have the size $A=\Delta \times \Delta$.

The model described in Eq. (3) represents the conventional near-field model, wherein the amplitude remains unchanged, but the phase changes according to the distance from each element of the RIS. On the other hand, the model presented in Eq. (4) corresponds to an enhanced near-field model, as proposed in^26^. In this improved model, the element’s amplitude is determined by its relative position to the location of the user, while the phase remains unchanged, as in Eq. (3). In comparison to the conventional and enhanced near-field channels, the phase in the far-field channel is given by $[{\mathcal {V}}({p})]_n=\exp \left( -j {\mathcal {E}}_n^{\top } {k}(\theta , \varphi )\right)$, while the amplitude remains unchanged for all RIS elements ${\Gamma }({p})=\Gamma \textbf{1}_N$.

**Figure 2:**
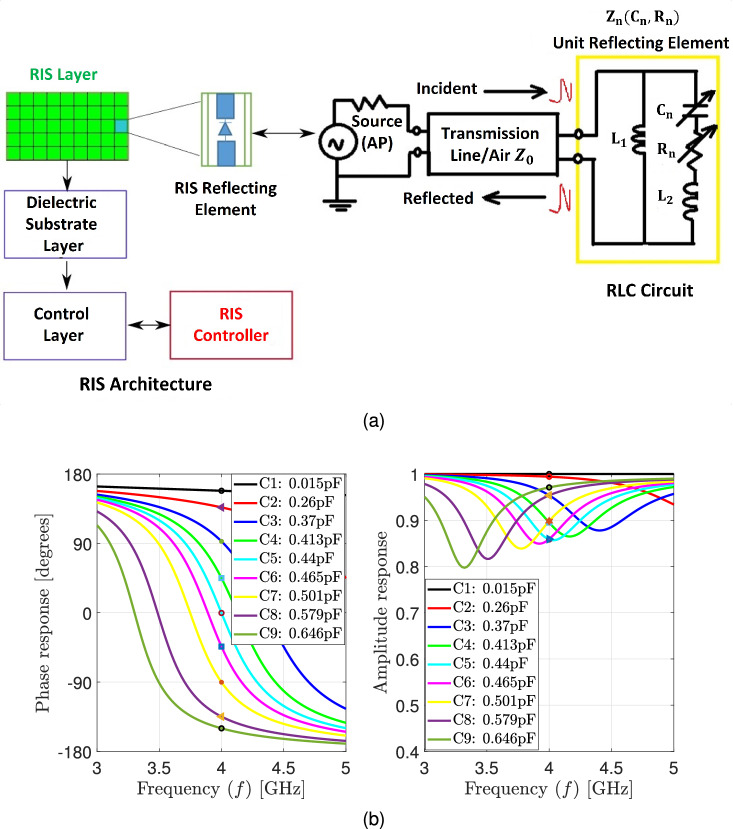
(**a**) Structure of the RIS including its reflecting element and the equivalent RLC circuit model and (**b**) amplitude and phase responses for different elements and their corresponding capacitance values.

**Figure 3:**
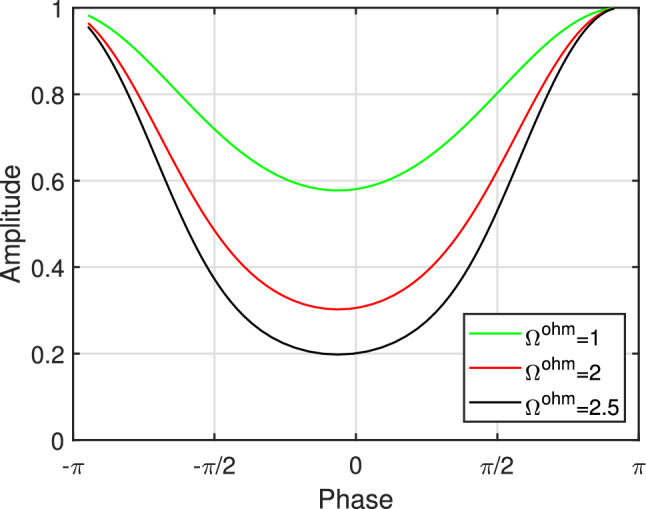
Reflected amplitude vs. phase shift for RIS element.

### RIS phase shift model

#### Amplitude variations

We adopt the practical model proposed in^30^ which describes the fundamental relationship between the reflection amplitude and the phase shift of the RIS. Consequently, we examine the near-field localization aided by RIS by incorporating an actual RIS amplitude model, which is based on the corresponding circuit setup of single RIS elements. In Fig. 2a, the equivalent model for the *n*-th reflecting element is illustrated as a parallel resonant circuit and its impedance is given by^30^:

5 $$\begin{aligned} \mathbb {Z}_{n}\left( C_{n}, \Omega _{n}^{\textrm{ohm}} \right) =\frac{j 2 \pi f I_{1}\left( j 2 \pi f I_{2}+\frac{1}{j 2 \pi f c_{n}}+\Omega _{n}^{\textrm{ohm}} \right) }{j 2 \pi f I_{1}+\left( j 2 \pi f I_{2}+\frac{1}{j 2 \pi f c_{n}}+\Omega _{n}^{\textrm{ohm}} \right) }. \end{aligned}$$

The bottom layer inductance, top layer inductance, effective capacitance, effective resistance, and carrier frequency of the incident signal are represented as $I_{1}, I_{2}, C_{n}, \Omega _{n}^{\textrm{ohm}}$ and *f* respectively. The reflection coefficient $v_{t,n}$ describes the portion of the reflected electromagnetic wave that is attributable to the discontinuity in impedance between the element impedance $\mathbb {Z}_{n}\left( C_{n}, \Omega _{n}^{\textrm{ohm}} \right)$ and free space impedance $\mathbb {Z}_{o}$:

6 $$\begin{aligned} v_{t,n}=\frac{\mathbb {Z}_{n}\left( C_{n}, \Omega _{n}^{\textrm{ohm}} \right) -\mathbb {Z}_{o}}{\mathbb {Z}_{n}\left( C_{n}, \Omega _{n}^{\textrm{ohm}} \right) +\mathbb {Z}_{o}}. \end{aligned}$$

$v_{t,n}$ being a function of $C_{n}, \Omega _{n}^{\textrm{ohm}}$ and *f*, allows us to control and programme the reflected electromagnetic waves by changing the values of $C_{n}, \Omega _{n}^{\textrm{ohm}}$ and *f*. $C_{n}$ has values that vary from 0.15 pF to 1.5 pF, $\Omega _{n}^{\textrm{ohm}} =1$ ohm, $\mathbb {Z}_{o}=377$, and $f=4 \textrm{GHz}$. The RIS element will scatter a sinusoidal signal impinging at frequency *f* with an amplitude of $\left| v_{t,n}\right|$ and a phase shift of $\arg \left( v_{t,n}\right)$. For example, in the case of a one-bit RIS, one positive-intrinsic-negative (PIN) diode is required per RIS element, and more diodes are needed for more resolution but at the cost of complex design. Consequently, the RIS is adjusted by using multiple PIN diodes numbers assigned to every element. Two alternative capacitance values can be used with each PIN diode. Figure 2b illustrates the responses of amplitude and phase for different values of capacitances. It was found that because the amplitude response and phase shifts of the reflecting element are typically non-linearly linked, they cannot be controlled separately. The reflection amplitude , as illustrated in Fig. 3, achieves a modest value at phase shift equals zero, but it grows consistently as the phase shift reaches $180^\circ$ or $-180^\circ$ and asymptotically approaches one. As a consequence, it is incorrect for many earlier research to assume that the amplitude response value is one. The reflecting phase shift provided by each capacitance value varies. For instance, the capacitance values of 0.5011 pF and 0.3732 pF correspond to phases of $-90^\circ$ and $90^\circ$ respectively, and will provide a $180^\circ$ phase shift spacing per element. Figure 2b shows the different values of the phase and amplitude responses with their corresponding capacitance values at center frequency $f=4\, \textrm{GHz}$.

The actual model considers variations in the amplitude, which are, in turn, determined by the phase, contrary to the commonly held assumption in the literature, which assumes a constant amplitude. More specifically, the performance loss in localization resulting from the discrepancy between the ideal and the actual model responses will be explored in this work. In the term, $\textbf{v}_t={\text {diag}}\left( v_{t, 0} \ldots v_{t, N-1}\right)$, let $v_{t, n}=\beta _{t, n}\left( \Theta _{t, n}\right) e^{j \Theta _{t, n}}$ where, $\Theta _{t, n} \in [-\pi , \pi )$ is the phase shift and $\beta _{t, n}\left( \Theta _{t, n}\right) \in [0,1]$ is the amplitude. The amplitude as a function of the phase can be represented as:

7 $$\begin{aligned} \beta _{t, n}\left( \Theta _{t, n}\right) =\left( 1-\beta _{\text{ min } }\right) \left( \frac{\sin \left( \Theta _n-\phi \right) +1}{2}\right) ^\gamma +\beta _{\text{ min } }. \end{aligned}$$

**Figure 4:**
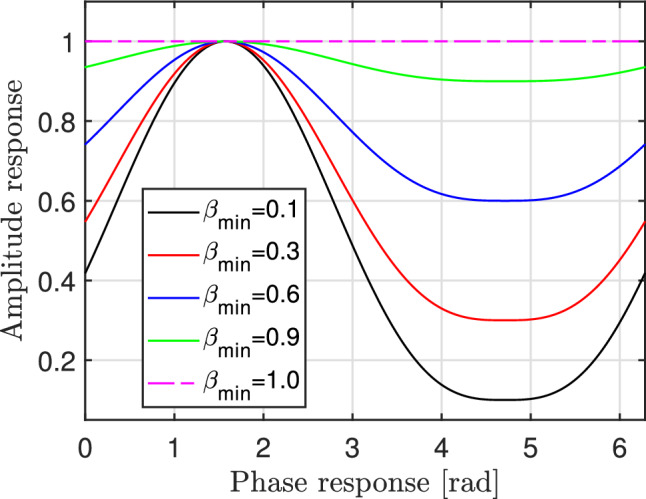
Amplitude variations for different values of $\beta _{\min }$, $\gamma =0$ and $\phi =1.5$.

The constants $\beta _{\min } \ge 0$, $\phi \ge 0$, and $\gamma \ge 0$, are parameters associated with the particular circuit implementation being considered. It should be noted that when $\beta _{\min }=1$ (or $\gamma =0$), Eq. (7) is essentially the same as the ideal phase shift model with unity amplitude. Figure 4 shows the amplitude variations for different values of $\beta _{\min }$. It is evident from Fig. 4 that when $\beta _{\min }=1$, the amplitude response is one while, it fluctuates between zero and one when $\beta _{\min }<1$.

Using this realistic model, we have designed the RIS phase profiles for directional and positional channel models. A design of phase profile is provided next.

#### Design of phase profile

**Figure 5:**
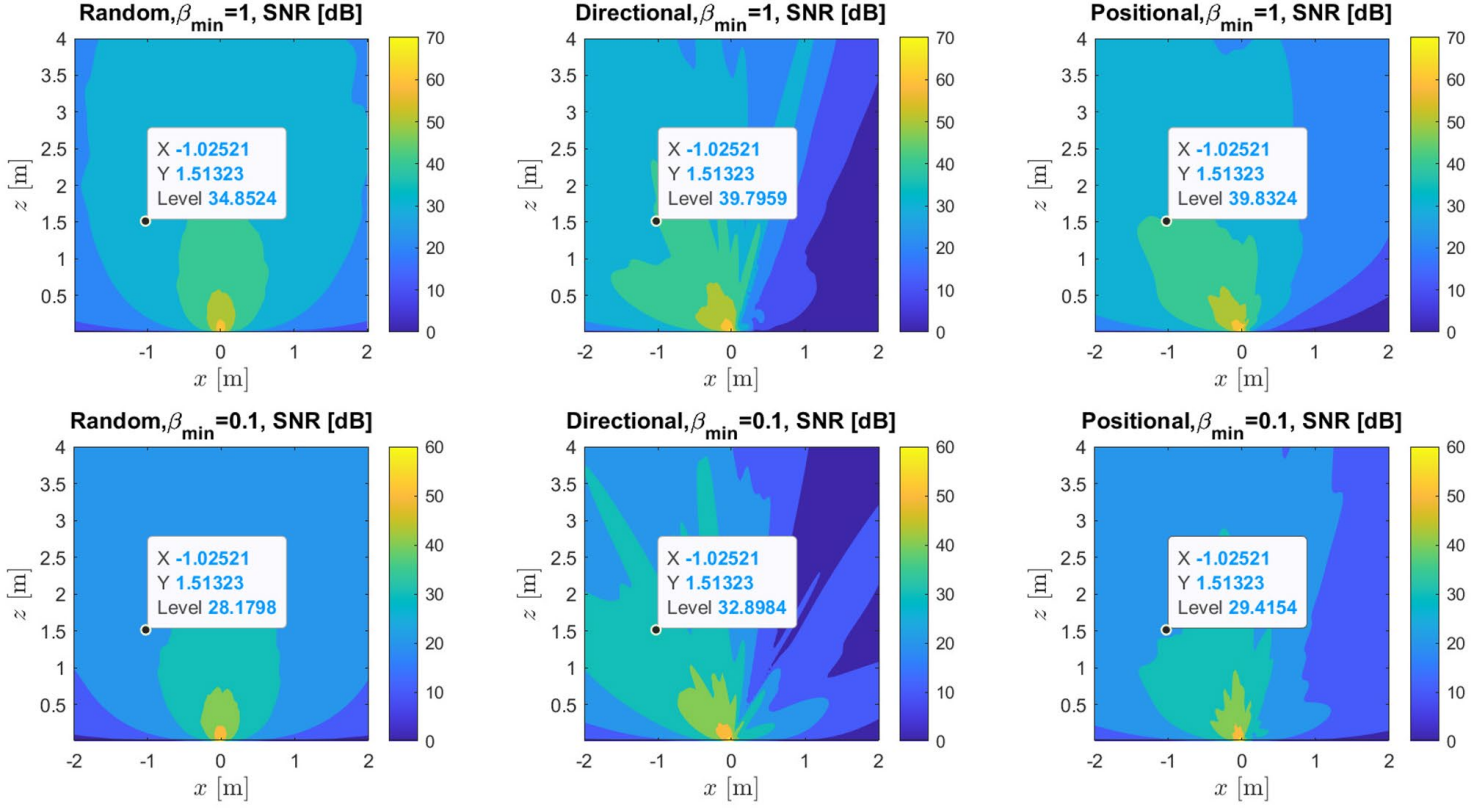
SNR in dB for random, directional and positional phase profile at $\beta _{\text{ min } }=\{0.1,1\}$.

The accuracy of determining the user location relies on the selection of the profiles of the RIS phases $\textbf{v}_t$, while the amplitudes are not assumed to be one as mentioned widely in the literature, however, they are distributed from [0, 1]. To eliminate the impact of phases in the channel ${h}_{\rm{ant}}$, we assign $\textbf{v}_t=\textbf{v}_{\rm{ant}} \tilde{\textbf{v}}_t$, where the known phases $\textbf{v}_{\rm{ant}}={\text {diag}}\left( u_{\rm{ant}, 0}, \ldots , u_{\rm{ant}, N-1}\right)$ are chosen such that $\left[ {h}_{\rm{ant}}^{\top } \textbf{v}_{\rm{ant}}\right] _n=\left| \left[ {h}_{\rm{ant}}^{\top }\right] _n\right| , \forall n$^31^. Where $u_{\text {ant},n}=\left[ \left( {h}_{\text{ ant } } /\left| {h}_{\text{ ant } }\right| \right) ^*\right] _n$. This approach leverages the information about ${h}_{\rm{ant}}$ to ensure the desired outcome. We are now left with the task of designing $\tilde{\textbf{v}}_t={\text {diag}}\left( \tilde{v}_{t, 0}, \ldots , \tilde{v}_{t, N-1}\right)$. We examine three configurations for $\tilde{v}_{t, n}, \forall n, t$. Firstly for the random configuration, we assign $\tilde{v}_{t, n}=\exp \left( j \Psi _{t, n}\right)$ where, $\Psi _{t, n} \sim \mathcal {U}(0,2 \pi )$ are independently and randomly distributed for every $n^\text {th}$ RIS element and $t\text {th}$ time instant. Secondly, we set the directional phase configuration as $\tilde{v}_{t, n}=\exp \left( +j {\mathcal {E}}_n^{\top } {k}\left( \theta ^{(k)}, \varphi ^{(k)}\right) \right)$. The priori PDF *f*(*p*) is used to generate the phase samples of $\theta ^{(k)}$ and $\varphi ^{(k)}$. Lastly, the positional phase configuration is obtained as $\tilde{v}_{t, n}=\exp \left( +j \frac{2 \pi }{\lambda }\left( \left\| {p}^{(k)}-\mathcal {{E}}_n\right\| -\mathcal {Q}^{(k)}\right) \right)$. We use the *f*(*p*) to extract the position ${p}^{(k)}$ and the distance $\mathcal {Q}^{(k)}$ samples. To comprehend the difference among various phase profiles and demonstrate the impact of the actual phase shift model outlined in Eq. (7), we illustrate SNR as a function of the location for a single realization of the three phase profiles as per Fig. 5. We assume a priori position distribution with mean $\mu _p=[-0.1,-0.1,-0.1]^{\top } \in \mathbb {R}^3$ and covariance ${c}_p=0.01 {I}_3 \in \mathbb {R}^{3 \times 3}$. Figure 5 shows the random, directional, and positional SNR for different values of $\beta _{\text{ min } }=\{0.1,1\}$. Generally, in the random scenario, we notice that the SNR remains consistent across all locations with decreased SNR near the outer edges of the RIS subject to the pathloss. However, higher SNR is attained in the selected direction $[-0.1,-0.1,-0.1]^{\top } \in \mathbb {R}^3$ for both directional and positional cases with decreased SNR in the other areas compared to the random scenario. This phenomenon can be elucidated in the following manner: the directional and positional phase profiles concentrate energy toward the user’s direction, resulting in a value of $\left\| {U}^{\top } {\mathcal {V}}({p})\right\| ^2 \approx 0$ approximately equal to 0 for the majority of locations *p* that deviate from the actual user location. Consequently, tuning the RIS phase profile will improves the PEB and the localization accuracy. Furthermore, when $\beta _{\min }=0.1$, the SNR degrades in all three scenarios in comparison with the case of the ideal lossless model $\beta _{\min }=1$. The SNR dB levels at the location (-1,1.5) is shown for different values of $\beta _{\text{ min } }$ for the random, directional and positional cases. The SNR can be given as follows:

8 $$\begin{aligned} \textrm{SNR}=\frac{1}{T} \sum _{t=1}^T \frac{E_s \Gamma ^2}{N_0}\left| {u}_t^{\top } {\mathcal {V}}({p})\right| ^2. \end{aligned}$$

### Problem statement

Our objective is to develop the PEB and create a simplified algorithm for the estimation of the user position *p*, along with the unknown channel gain ${\Gamma }$, based on the *T* transmission instances and the observations $r_t$ mentioned in Eq. (1). We assume that the practical model in Eq. (5) and its associated RIS parameters, $\beta _{\min }$, $\phi$, and $\gamma$ are known. In this paper, we put the assumption that these parameters are available for the case of the uplink single-user scenario when the RIS act as lens^21^. In addition to localization and unlike the widely used far-field model in the literature, we have investigated the effect of the practical phase shift model on the achievable data rate in the near-field regime taking into consideration the random, directional and positional RIS phase profiles.

## Fisher information matrix (FIM) analysis

Considering the practical phase shift model in “System model”, the baseband observations at the receiver for the transmitted pilots after time *t* in the near field regime can be rewritten using Eqs. (1) and (2) as:

9 $$\begin{aligned} r_t=\Gamma e^{j \vartheta } \sum _{n=1}^N[{\mathcal {V}}({p})]_n {u}_{t, n} \mathcal {S}_t+\mathcal {Z}_t, \end{aligned}$$

where, ${u}_{t, n}=v_{t, n}\left[ {h}_{a n t}\right] _n=\left( \beta _{t, n}\left( \Theta _{t, n}\right) e^{j \Theta _{t, n}}\right) \left[ {h}_{a n t}\right] _n$ and the amplitude $\beta _{t, n}\left( \Theta _{t, n}\right)$ is defined in (7). The model in (9) can be represented in a vector form.

10 $$\begin{aligned} {r}=\sqrt{E_s} \Gamma e^{j \vartheta } {U}^{\top }({\Omega }) {\mathcal {V}}({p})+{\mathcal {Z}}, \end{aligned}$$

where, ${U}({\Omega })=\left[ {u_1}({\Omega }) \ldots {u_T}({\Omega })\right] \in \mathbb {C}^{N \times T}$ is the matrix of the RIS profile, depending on the values of the RIS amplitude circuit parameters $\Omega =\left[ \beta _{\text{ min } }, \phi , \gamma \right] ^{\top }$ in (7). Considering the noise free vector ${\xi }=\sqrt{E_s} \Gamma e^{j \vartheta } {U}^{\top }({\Omega }) {\mathcal {V}}({p})$ and the $8 \times 1$ vector of unknown parameters ${\Upsilon }=\left[ \Gamma , \vartheta , {p}^{\top }, \beta _{\min }, \phi , \gamma \right] ^{\top }$, the FIM ${J}({\Upsilon }) \in \mathbb {R}^{8 \times 8}$ is defined as^32^.

11 $$\begin{aligned} {J}({\Upsilon })=\frac{2}{N_0} \Re \left\{ \left( \frac{\partial {\xi }}{\partial {\Upsilon }}\right) ^{\textrm{H}} \frac{\partial {\xi }}{\partial {\Upsilon }}\right\} . \end{aligned}$$

The FIM diagonal and off-diagonal elements can be generated from the partial derivatives. However, we put the assumption that the RIS phase profile and the related amplitude circuit parameters are known at the receiver so, the $8 \times 1$ vector of unknown parameters ${\Upsilon }=\left[ \Gamma , \vartheta , {p}^{\top }, \beta _{\min }, \phi , \gamma \right] ^{\top }$ can be reduced to ${\Upsilon }=\left[ \Gamma , \vartheta , {p}^{\top }\right] ^{\top }$. We can represent (11) as follows:

12 $$\begin{aligned} {J}({\Upsilon })=\left[ \begin{array}{lll} {J}_{\Gamma , \Gamma } &{} {J}_{\Gamma , \vartheta } &{} {J}_{\Gamma , p} \\ {J}_{\vartheta , \Gamma } &{} {J}_{\vartheta , \vartheta } &{} {J}_{\vartheta , p} \\ {J}_{p, \Gamma } &{} {J}_{p, \vartheta } &{} {J}_{p, p} \end{array}\right] , \end{aligned}$$

where, ${J}_{\Gamma , \Gamma } \triangleq [{J}({\Upsilon })]_{1,1}$ is the first diagonal element of ${J}({\Upsilon })$, ${J}_{\vartheta , \vartheta } \triangleq [{J}({\Upsilon })]_{2,2}$ is the second diagonal element and ${J}_{p, p} \triangleq [{J}({\Upsilon })]_{3: 5,3: 5}$ are the third, fourth and fifth diagonal elements of ${J}({\Upsilon })$. The off-diagonal elements above and below the main diagonal are $$\left[ \begin{array}{ll}{J}_{\Gamma , p}&{J}_{\vartheta , p}\end{array}\right] ^{\top } \triangleq [{J}({\Upsilon })]_{1: 2,3: 5}$$ and $\left[ {J}_{p, \Gamma } \quad {J}_{p, \vartheta }\right] \triangleq [{J}({\Upsilon })]_{3: 5,1: 2}$ respectively. To determine the diagonal and off-diagonal elements of the FIM ${J}({\Upsilon })$, we need to calculate the partial derivatives $\partial {\xi } / \partial \Gamma =\sqrt{E_s} e^{j\vartheta } {U}^{\top } {\mathcal {V}}({p})$, $\partial {\xi } / \partial \vartheta =\sqrt{E_s} \Gamma j e^{j \vartheta } {U}^{\top } {\mathcal {V}}({p})$ and $\partial {\xi } / \partial {p}=\sqrt{E_s} \Gamma e^{j\vartheta } {U}^{\top } {F}({p})$, where ${F}({p}) \triangleq \frac{\partial {\mathcal {V}}({p})}{\partial {p}}=j \frac{2 \pi }{\lambda }\left( {\text {diag}}({\mathcal {V}}({p})) {W}^{\top }+{\mathcal {V}}({p}) \frac{{p}^{\top }}{d}\right) \in \mathbb {C}^{N \times 3}$ and ${W}=\left[ {w_0},{w_1},\ldots , {w_{N-1}}\right]$ with ${w_n}=\left( \mathcal {E}_n-{p}\right) /\left\| \mathcal {E}_n-{p}\right\|$. Consequently, the elements inside the FIM can be written as follows:

13 $$\begin{aligned} {J}({\Upsilon })=\frac{2 E_s}{N_0}\left[ \begin{array}{ccc} {\mathcal {V}}^{\textrm{H}}({p}) {\Xi } {\mathcal {V}}({p}) &{} {\Gamma } {\mathcal {V}}^{\textrm{H}}({p}) {\Xi } {\mathcal {V}}({p}) &{} {\Gamma } \Re \left( {\mathcal {V}}^{\textrm{H}}({p}) {\Xi } {F}({p})\right) \\ {\Gamma }\left( {\mathcal {V}}^{\textrm{H}}({p}) {\Xi } {\mathcal {V}}({p})\right) &{} {\Gamma }^2 {\mathcal {V}}^{\textrm{H}}({p}) {\Xi } {\mathcal {V}}({p}) &{} {\Gamma }^2 \mathfrak {I}\left( {\mathcal {V}}^{\textrm{H}}({p}) {\Xi } {F}({p})\right) \\ {\Gamma } \Re \left( {\mathcal {V}}^{\textrm{H}}({p}) {\Xi } {F}({p})\right) &{} {\Gamma }^2 \mathfrak {J}\left( {\mathcal {V}}^{\textrm{H}}({p}) {\Xi } {F}({p})\right) &{} {\Gamma }^2 \mathfrak {R}\left( {F}^{\textrm{H}}({p}) {\Xi } {F}({p})\right) \end{array}\right] , \end{aligned}$$

where $\Re (.)$ and $\Im (.)$ are the real and imaginary of a complex number and the positive semidefinite matrix ${\Xi }={U}^* {U}^{\top }$.

Definition 1 Equivalent FIM (EFIM): Given a parameter vector ${\Phi } \triangleq \left[ {\Phi }_1^{\top }, {\Phi }_2^{\top }\right] ^{\top }$ with related FIM.

14 $$\begin{aligned} \textbf{J}_{{\Phi }}=\left[ \begin{array}{cc} \textbf{J}_{{\Phi }_1} &{} \textbf{J}_{{\Phi }_1 {\Phi }_2} \\ \textbf{J}_{{\Phi }_1 {\Phi }_2} &{} \textbf{J}_{{\Phi }_2} \end{array}\right] . \end{aligned}$$

Then, the EFIM of $\Phi _1$ is given by Schur complement as^33,34^

15 $$\begin{aligned} \textbf{J}_{{\Phi }_1}^{\textrm{e}}=\textbf{J}_{{\Phi }_1}-\textbf{J}_{{\Phi }_1 {\Phi }_2} \textbf{J}_{{\Phi }_2}^{-1} \textbf{J}_{{\Phi }_1 {\Phi }_2}^{\top }. \end{aligned}$$

As per this definition, it should be noted that $\textbf{J}_{{\Phi }_1}$ is the FIM of $\Phi _1$ under the assumption that $\Phi _2$ is known. Additionally, $\textbf{J}_{{\Phi }_1 {\Phi }_2} \textbf{J}_{{\Phi }_2}^{-1} \textbf{J}_{{\Phi }_1 {\Phi }_2}^{\top }$ quantifies the amount of information lost due to the uncertainty surrounding $\Phi _2$. Taking into account Definition 1, the equivalent FIM of the user position is given as:

16 $$\begin{aligned} \begin{aligned} {J}({p})&=[{J}({\Upsilon })]_{3: 5,3: 5}-[{J}({\Upsilon })]_{3: 5,1: 2}[{J}({\Upsilon })]_{1: 2,1: 2}^{-1}[{J}({\Upsilon })]_{1: 2,3: 5} \\&=\frac{2 \Gamma ^2 E_s}{N_0} \Re \left\{ {F}^{\textrm{H}}({p})\left[ {\Xi }-\frac{{\Xi } {\mathcal {V}}({p}) {\mathcal {V}}^{\textrm{H}}({p}) {\Xi }}{{\mathcal {V}}^{\textrm{H}}({p}) {\Xi }({p})}\right] {F}({p})\right\} . \end{aligned} \end{aligned}$$

Consequently, the PEB is represented as:

17 $$\begin{aligned} {\text {PEB}}=\sqrt{{\text {trace}}\left( \left[ {J}^{-1}({\Upsilon })\right] _{3: 5,3: 5}\right) }. \end{aligned}$$

The RMSE of any unbiased estimator $\hat{{p}}$ can be bounded by an inequality:

18 $${\textrm{PEB}} \le {\textrm{RMSE}} \triangleq \sqrt{{\mathbb {E}} \left\{ \Vert {\hat{p}}-{p}\Vert ^2\right\} }. $$

## Low complexity estimation

### Maximum likelihood (ML) estimation

The joint PDF can be expressed as^32,35^:

19 $$\begin{aligned} f({r} \mid {\Upsilon })=\left( \frac{1}{\pi N_0}\right) ^T \exp \left( -\frac{1}{N_0}\Vert {r}-{\xi }({\Upsilon })\Vert ^2\right) , \end{aligned}$$

where the noise free term ${\xi }({\Upsilon })=\mathcal {G} \sum _{n=1}^N[{\mathcal {V}}({p})]_n {u}_{t, n} \mathcal {S}_t$, $\mathcal {G}=\Gamma e^{j \vartheta }$. Consequently, the ML estimate of the channel gain and user location:

20 $$\begin{aligned} \begin{aligned} {[\hat{\mathcal {G}}, \hat{{p}}] }&=\arg \max _{\mathcal {G}, {p}} f({r} \mid \mathcal {G}, {p}) \\&=\arg \min _{\mathcal {G}, {p}} \underbrace{\left\| {r}-\sqrt{E_s} \mathcal {G} {U}^{\top } {\mathcal {V}}({p})\right\| ^2}_{{d}(\mathcal {G}, p)} \end{aligned}. \end{aligned}$$

Differentiating the term ${d}(\mathcal {G}, p)$ and set equal to zero, yields to the estimate that maximize the likelihood function $\frac{\partial {d}(\mathcal {G}, p)}{\partial \mathcal {G}}=0$. Consequently, solving for $\mathcal {G}$ will lead to the estimate as a function of *p*.

21 $$\begin{aligned} \hat{\mathcal {G}}({p})=\frac{{\mathcal {V}}^{\textrm{H}}({p}) {U}^* {r}}{\sqrt{E_s}\left\| {U}^{\top } {\mathcal {V}}({p})\right\| ^2}. \end{aligned}$$

Therefore, the estimate *p* can be computed as:

22 $$\begin{aligned} \hat{{p}}=\arg \min _{{p}}\left\| {r}-\sqrt{E_s} \hat{\mathcal {G}}({p}) {U}^{\top } {\mathcal {V}}({p})\right\| ^2. \end{aligned}$$

### Simple user localization

The framework of the user localization problem in spherical coordinates is used to solve (22). This framework gives rise to a three-step estimation process outlined below. Initially, we represent each exponential term in the far-field, ${\mathcal {V}}(\varphi , \theta )=\left[ e^{-\textrm{j} {k}(\varphi , \theta )^{\top } \mathcal {{E}}_1}, \ldots , e^{-\textrm{j} {k}(\varphi , \theta )^{\top } \mathcal {E}_N}\right]$ as $[{\mathcal {V}}(\varphi , \theta )]_n=\exp \left( -j \frac{2 \pi }{\lambda } \ell _n \sin (\theta ) \cos \left( \varphi -\Psi _n\right) \right)$. We utilize Jacobi-Anger expansion method^21^ to express $[{\mathcal {V}}(\varphi , \theta )]_n$ as:

23 $$\begin{aligned}{}[{\mathcal {V}}(\theta , \varphi )]_n \approx \sum _{m=-L}^L j^m \mathcal {J}_m\left( -\frac{2 \pi }{\lambda } \ell _n \sin (\theta )\right) e^{j m\left( \varphi -\Psi _n\right) }, \end{aligned}$$

where $L>\frac{2 \pi }{\lambda } \ell _{\max } \sin \theta$ is the number of terms in the expansion that gives sufficient precision for high-quality approximation. Consequently, $T \ge T_{\rm{thr}}=2 L+1$ is adopted for low complexity solution as per Algorithm 1. Furthermore, $\mathcal {J}_m(\cdot )$ is the *m*-th order Bessel function of the first kind. The expansion in (23) can be represented as:

24 $$\begin{aligned}{}[{\mathcal {V}}(\theta , \varphi )]_n={g}_n^{\top }(\theta ) {h}(\varphi ), \end{aligned}$$

where $\left[ {g}_n(\theta )\right] _m=j^m \mathcal {J}_m\left( -\frac{2 \pi }{\lambda } \ell _n \sin (\theta )\right) e^{-j m \Psi _n}$ and $[{h}(\varphi )]_m=e^{j m \varphi }$. It can be readily confirmed that ${\mathcal {V}}(\theta , \varphi ) \approx {G}^{\top }(\theta ) {h}(\varphi )$, where ${G}(\theta )=\left[ {g}_0(\theta ) \ldots {g}_{N-1}(\theta )\right]$. Subsequently, the response vector ${\mathcal {V}}(\theta , \varphi )$ has independent structure in the angles $\theta$ and $\varphi$. We begin with three-stage estimating method to estimate $\hat{{p}}={p}(\hat{\mathcal {Q}}, \hat{\theta }, \hat{\varphi })$. We rewrite the system in (10) as:

25 $$\begin{aligned} {r}=\sqrt{E_s} \mathcal {G} {U}^{\top } {G}^{\top }(\theta ) {h}(\varphi )+{\mathcal {Z}}. \end{aligned}$$

By defining the vector *b* as ${b}=\sqrt{E}_s\mathcal {G} {h}(\varphi )$ based on (25), the estimation of *b* as a function of $\theta$ can be represented by $\hat{{b}}(\theta )=\left( \left( {U}^{\top } {G}^{\top }(\theta )\right) ^{\top } {U}^{\top } {G}^{\top }(\theta )\right) ^{-1}\left( {U}^{\top } {G}^{\top }(\theta )\right) ^{\top } {r}$. Therefore, the estimation for the angle $\theta$ can be applied as:

26 $$\begin{aligned} \hat{\theta }=\arg \min _\theta \left\| {r}-{U}^{\top } {G}^{\top }(\theta ) \hat{{b}}(\theta )\right\| ^2. \end{aligned}$$

Using the estimated value $\hat{\theta }$, (23) can be rewritten as:

27 $$\begin{aligned} {r}=\sqrt{E_s} \mathcal {G} {U}^{\top } {G}^{\top }(\hat{\theta }) {h}(\varphi )+{\mathcal {Z}}. \end{aligned}$$

For each value of $\varphi$, we estimate $\mathcal {G}$ in a similar manner as in (21), but instead of using ${\mathcal {V}}({p})$, we substitute it with ${G}^{\top }(\hat{\theta }) {h}(\varphi )$, resulting in the estimation $\hat{\mathcal {G}}(\varphi )$

28 $$\begin{aligned} \hat{\mathcal {G}}(\varphi )=\frac{{h}(\varphi ) {G}^*(\hat{\theta }) {U}^* {r}}{\sqrt{E_s\left\| {U}^{\top } {G}^{\top }(\hat{\theta }) {h}(\varphi )\right\| ^2}}. \end{aligned}$$

Therefore, we solve for $\varphi$:

29 $$\begin{aligned} \hat{\varphi }=\arg \min _{\varphi }\left\| {r}-\sqrt{E}_s \hat{\mathcal {G}}(\varphi ) {U}^{\top } {G}^{\top }(\hat{\theta }) {h}(\varphi )\right\| ^2. \end{aligned}$$

With the estimated angles $\hat{\theta }$ and $\hat{\varphi }$, we define $${p}(\mathcal {Q})=\mathcal {Q}\left[ \begin{array}{lll}\sin \hat{\theta } \cos \hat{\varphi }&\sin \hat{\theta } \sin \hat{\varphi }&\cos \hat{\theta }\end{array}\right] ^{\top }$$, which allows us to determine $\hat{\mathcal {G}}({p}(\mathcal {Q}))$, as described in (19). Finally, we proceed to solve the optimization problem.

30 $$\begin{aligned} \hat{\mathcal {Q}}=\arg \min _{\mathcal {Q}}\left\| {r}-\sqrt{E_s} \hat{\mathcal {G}}({p}(\mathcal {Q})) {U}^{\top } {\mathcal {V}}({p}(\mathcal {Q}))\right\| ^2. \end{aligned}$$

Now we can calculate the user estimate as:

31 $$\begin{aligned} \hat{{p}}=\hat{\mathcal {Q}}\left[ \begin{array}{lll} \sin \hat{\theta } \cos \hat{\varphi }&\sin \hat{\theta } \sin \hat{\varphi }&\cos \hat{\theta } \end{array}\right] ^{\top }. \end{aligned}$$

Algorithm 1 summarizes the steps of estimating the user localization.

### Computational complexity

To assess the computational complexity of Algorithm 1, consider discretizing the search intervals for distance, azimuth, and elevation into grids with a size of $\Delta$ each^20^. We make the assumption that both $\Delta$ and *N* are greater than *T*, which is a reasonable assumption considering the substantial size of the RIS and the need for a detailed search granularity to achieve high-quality estimates. Initially, assume that $T<T_{t h r}$. It is evident that the complexity of Algorithm 1 is primarily influenced by the 2D search process used to estimate azimuth and elevation angles. In the algorithm, it is satisfactory to focus on analyzing the computational complexity of the 2D search outlined in (22). Let us denote $\mathcal {F}(\theta , \varphi )=\hat{\mathcal {G}}(p) U^{\top } \mathcal {V}(p)$ so, the complexity of $\mathcal {F}(\theta , \varphi )$ is $\mathcal {O}(T N)$. The estimation problem in (22) is equivalent to the following problem:

32 $$\begin{aligned} (\hat{\theta }, \hat{\varphi })=\underset{\theta , \varphi }{\arg \max } \frac{\mathcal {F}(\theta , \varphi )^{\textrm{H}} r}{\Vert \mathcal {F}(\theta , \varphi )\Vert ^2}. \end{aligned}$$

**Figure 6:**
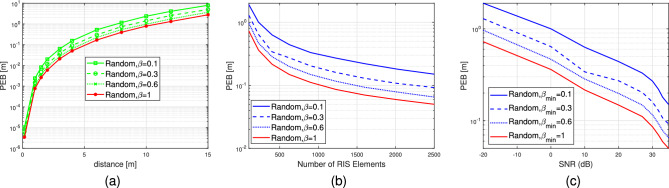
(**a**) PEB as a function of distance to the RIS lens, $\beta _{\min } \in \{0.1,0.3,0.6,1\}$. (**b**) PEB as a function of number of RIS elements. (**c**) PEB versus SNR (dB).

**Figure 7:**
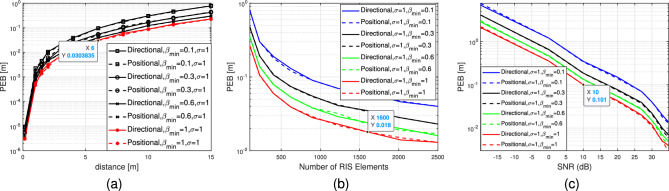
(**a**) PEB as a function of distance to the RIS lens,$\sigma =1$, and $\beta _{\min } \in \{0.1,0.3,0.6,1\}$. (**b**) PEB as a function of Number of RIS elements $\sigma =1$, and $\beta _{\min } \in \{0.1,0.3,0.6,1\}$. (**c**) PEB versus SNR (dB) $\sigma =1$, and $\beta _{\min } \in \{0.1,0.3,0.6,1\}$.

Consequently, following the computation of $\mathcal {F}(\theta , \varphi )$, a search is required over both $\theta$ and $\varphi$. Therefore, the overall computational cost of Algorithm 1 when $T \le T_{t h r}$ is $\mathcal {O}\left( T \Delta ^2 N\right)$. When $T \ge T_{t h r}$, the complexity of Algorithm 1 is primarily influenced by the estimation of $\theta$. To estimate $\theta$, the initial step involves computing $\mathcal {F}(\theta ) \triangleq G^{\top }(\theta )$ where, $G(\theta )=\left[ g_0(\theta ) \ldots g_{N-1}(\theta )\right]$. The computational cost of $\mathcal {F}(\theta )$ is $\mathcal {O}(T N(2 L+1))$ since $T \ge T_{\rm{thr}}=2 L+1$ Subsequently, a search for $\theta$ within the range $[0, \pi / 2]$ is required so, the complexity will be $\mathcal {O}({\text {TN}}(2 L+1) \Delta )$. The comprehensive complexity of Algorithm 1 is expressed as $\mathcal {O}\left( T \Delta ^2 N\right)$ when $T \le T_{t h r}=2 L+1$ and $\mathcal {O}(T N(2 L+1) \Delta )$ when $T \ge T_{\rm{thr}}$. Consequently, it can be inferred that as *T* becomes sufficiently large, the adoption of the Jacobi-Anger expansion-based estimator in results in a reduction in computational complexity from $\mathcal {O}\left( \Delta ^2\right)$ to $\mathcal {O}(\Delta )$ and hence converting the ac2D search problem to one-dimensional (1D) simple search line. Moreover, the decrease in complexity has not compromised accuracy, which is maintained at the decimeter level, as illustrated in Figs. 6 and 7. This observation strongly suggests the efficacy of the high-quality and optimal estimator employed in the algorithm.

**Algorithm 1:**
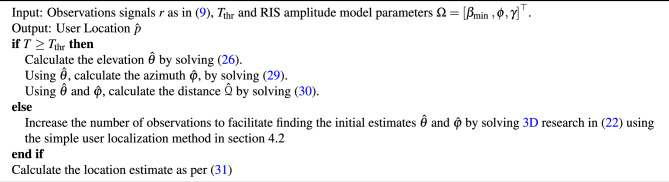
User location estimate ${p}(\hat{\mathcal {Q}}, \hat{\theta }, \hat{\varphi })$ in RIS-assisted near-field localization.

## Achievable data rate in the near field regime

Typically, the designs that aim to minimize the PEB differ from those that prioritize communication-related metrics like capacity optimization. While both types of designs exhibit improved performance in high SNR conditions, the accuracy of localization is also influenced by factors such as geometric considerations and the possibility of distinguishing signals from various paths rather than merely aligning them^5^. In this section of the paper, we study the performance loss on the achievable data rate in the near field channel when the realistic RIS phase profile is adopted. The achievable data rate can be represented as per^31^.

33 $$\begin{aligned} \begin{aligned} R_{\rm{RIS}}&=\max _{\Theta _{t, n}, \beta _{t, n}} \log _2(1+S N R) \\&=\log _2\left( 1+\frac{1}{T} \sum _{t=1}^T \frac{E_s \Gamma ^2}{N_0}\left| {{u}}_t^{\top } {\mathcal {V}}({p})\right| ^2\right) . \end{aligned} \end{aligned}$$

The maximum data rate is selected when the phase shifts are tuned. Recall that ${u}_{t, n}=v_{t, n}\left[ {h}_{a n t}\right] _n=\left( \beta _{t, n}\left( \Theta _{t, n}\right) e^{j \Theta _{t, n}}\right) \left[ {h}_{a n t}\right] _n$ for each $n^\text {th}$ element at time instant *t*. As a result, at the random case, we adopt $\Theta _{t, n}=\exp \left( j \Psi _{t, n}\right)$ where $\Psi _{t, n} \sim \mathcal {U}(0,2 \pi )$, while in both directional and positional scenarios, we respectively assign the phases $\Theta _{t, n}=\exp \left( +j \mathcal {{E}}_n^{\top } {k}\left( \theta ^{(k)}, \varphi ^{(k)}\right) \right)$ and $\Theta _{t, n}=\exp \left( +j \frac{2 \pi }{\lambda }\left( \left\| {p}^{(k)}-\mathcal {{E}}_n\right\| -\mathcal {Q}^{(k)}\right) \right)$. The amplitude varies as $\beta _{t, n}=\in (0,1]$.

**Figure 8:**
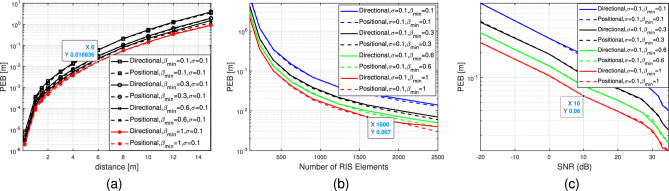
(**a**) PEB as a function of distance to the RIS lens,$\sigma =0.1$, and $\beta _{\min } \in \{0.1,0.3,0.6,1\}$. (**b**) PEB as a function of Number of RIS elements $\sigma =0.1$, and $\beta _{\min } \in \{0.1,0.3,0.6,1\}$. (**c**) PEB versus SNR (dB) $\sigma =0.1$, and $\beta _{\min } \in \{0.1,0.3,0.6,1\}$.

## Simulation results

**Figure 9:**
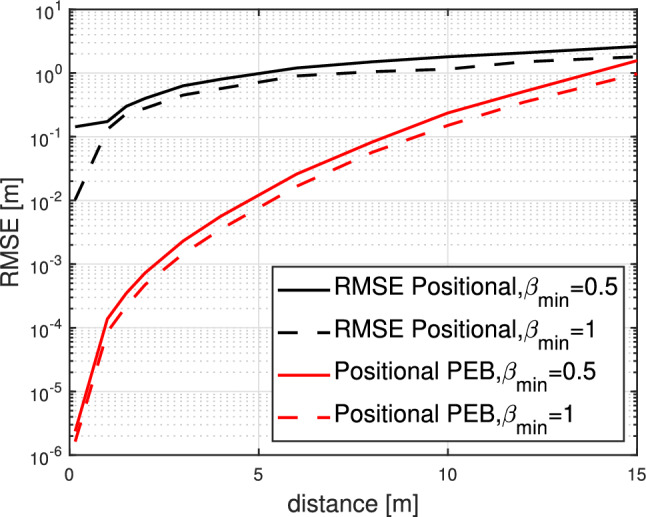
RMSE as a function of distance to the RIS lens, $\sigma =0.1$ and $\beta _{\min } \in \{0.5,1\}$.

We used the simulation parameters provided in^21^ but with the realistic RIS phase shift model as in “System model”, so, $\Omega =\left[ \beta _{\min }, \phi , \gamma \right] ^{\top }$. We select the range of values for $\beta _{\min } \in [0,1]$ and fixed the constants $\phi$ and $\gamma$ to 0 and 1.5 to study the performance loss not only on the localization but also in the communication. We examine a RIS composed of $N=2500$ elements arranged in $N_v \times N_h$ grid, where $N_v=N_h=50$, at a frequency of 28 GHz with $\lambda / 2$ and $\lambda ^2 / 4$ element spacing and area, respectively. The receive antenna is positioned at coordinates $[0,0,-\lambda ]^{\top }$. The transmit power, the noise power spectral density, and the reception noise figure are set to 1 mW, $-174$ dBm/Hz and 8 dB, respectively. The total pilot transmissions $T=200$ and the bandwidth is fixed to 1 MHz. In our analysis, we focus on a user who possesses a wavevector *k* oriented in the direction $[1,1,1]^{\top }$. The channel gains to and from the RIS are set according to Eqs. (3) and (4). The prior knowledge about the user’s location is represented by the PDF as $f({p})=\mathcal {N}\left( {p}; \mu _p, {c}_p\right)$, where ${c}_p=\sigma ^2 {I}_3$. It is solely employed for designing the directional beams of the RIS, and it is not utilized during the localization process or in the calculation of the PEB. The standard deviation in each dimension XYZ of the prior covariance of the user position is set to $\sigma \in \{0.1,1\}$ m to evaluate the localization performance in the random, directional, and optional phase profiles. In Eq. (21), the number of the terms in the expansion $L=5$ is used for low complexity estimation.

**Figure 10:**
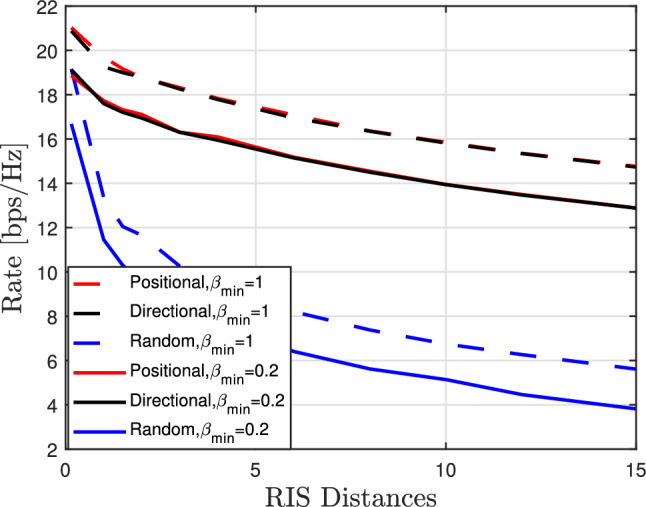
Data Rate versus RIS distance, $\beta _{\min } \in \{0.2,1\}$.

Figures 6,7 and 8 illustrate the PEB as a function of distance in meters, number of RIS elements and SNR (dB). The figures display the PEB for the three selected designs of the RIS phase profiles, i.e., random, directional and positional, considering various values of standard deviation $\sigma \in \{0.1,1\}$ and amplitude variation $\beta _{t, n}=\in (0,1]$. In Fig. 6a, it is noticed that by using a basic random phase configuration, we can achieve relatively low PEB, specifically below 1 meter, for user positions within a 10-meter distance from the RIS lens and at different values of $\beta _{\min }$. Figure 6b demonstrates the impact of varying RIS sizes on the PEB limit at different values of $\beta _{\min }$ when the UE distance is 5 meters. It is evident that for all the curves under consideration for different $\beta _{\min }$, the reduction in PEB is directly proportional to *N*. This relationship arises from the boost in SNR in the reflected path from the RIS to the receive antenna, which is directly proportional to the RIS’s different dimensional sizes *N*. Consequently, Fig. 6c shows the PEB performance in the low and high SNR regimes. Additionally, the absence of significant beamforming gain is attributed to the randomness of phase shifts. Figure 7 demonstrates that by employing directional or positional phase profiles ($\sigma =1$ and $\beta _{t, n}=\in (0,1]$), it is possible to significantly decrease the PEB. The positional phase profile exhibits slightly superior performance compared to the directional phase profile, although the disparity between the two is negligible. Similarly, in Fig. 7a, we observe that employing directional or positional phase profile allows us to achieve relatively enhanced PEB, specifically below 1 meter, for user positioned within a 10-meter range from the RIS lens, regardless of the value of $\beta _{\min }$. Figure 7b illustrates how the PEB limit is affected by varying RIS sizes at different $\beta _{\min }$ values when the distance between the user and the RIS is 5 meters. It is clear that for all the plotted curves corresponding to different $\beta _{\min }$ values, the reduction in PEB is directly proportional to *N*. This correlation stems from the boost in SNR in the reflected path, which is directly proportional to *N*, as depicted in Fig. 7c. Moreover, the substantial beamforming gain is ascribed to the directional and positional phase shifts directed towards the user’s location. Furthermore, the positional and directional phase profiles in Fig. 7 exhibit superior PEB performance compared to the random phase profile in Fig. 6 because of the tuned RIS coefficients that help the direct and the reflecting channel to be superimposed more constructively at the receive antenna. Similar to Figs. 7, 8 studies the PEB against distance from RIS, RIS different sizes and finally the SNR regimes but for a smaller value of standard deviation $\sigma$. Consequently, it is evident that having more precise a priori information (i.e., a smaller value for $\sigma =0.1$) results in improved PEB. For example, the PEB in Fig. 8a for the positional case, $\sigma =0.1$ and $\beta _{\min }=1$ at distance 6 m is 0.017 m however, the corresponding value in Fig. 7a at the same distance is 0.03 m. Furthermore, the PEB in Fig. 8b for the positional case, $\sigma =0.1$ and $\beta _{\min }=1$ at RIS number of elements 1600 is 0.007 m while its corresponding value in Fig. 7b is 0.018 m. the same study can be compared between Fig. 8c and Fig. 7c for the SNR scenario. The PEB is 0.101 at 10 dB when the standard deviation $\sigma =1$, while it is 0.06 when $\sigma =0.1$ as illustrated in Figs. 7c and Fig. 8c respectively.

**Figure 11:**
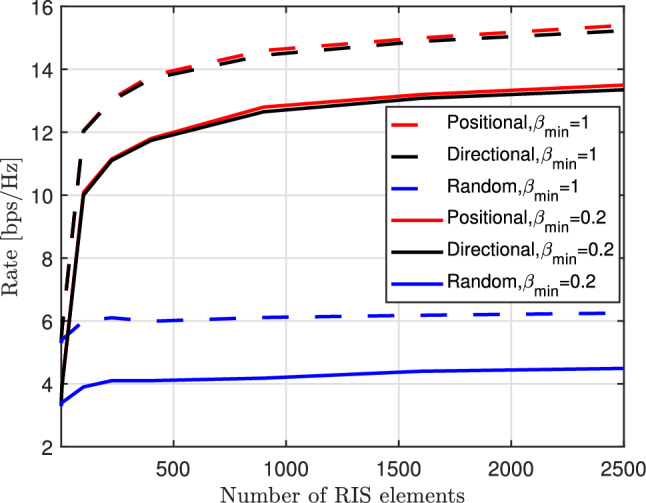
Data rate versus number of RIS elements, $\beta _{\min } \in \{0.2,1\}$.

Figure 9 illustrates the RMSE of three-stage localization algorithm, plotted against the distance from the RIS in meters. We notice how the RMSE performance is affected by adopting the true RIS phase shift model considering $\beta _{\min }=0.5$. Due to the adoption of a definite limit of the angle and delay resolutions (360 bins for $\varphi$, 90 for $\theta$, and 1000 for $\mathcal {Q}$), coupled with the limitation that the far-field presumed in the initial stages of the algorithm is not applicable for small distances, it becomes impractical to achieve the desired RMSE in the far-field regime. However, considering these factors, the performance of the positional RMSE remains far from the positional PEB, resulting in a performance that is significantly worse than what was forecasted by the bounds. This phenomenon can be justified by the following: The positional phase profiles concentrate energy towards the user’s direction, resulting in the majority of locations, apart from the true location, having a value of $\left\| {U}^{\top } {\mathcal {V}}({p})\right\| ^2 \approx 0$. Consequently, the objective function (22) remains nearly consistent across various positions, displaying very slender peaks around the actual position. Due to the limited precision of the estimators, there is a high probability of missing this narrow peak, which can result in a deteriorated positional RMSE.

Figure 10 shows the data rate against the distance to the RIS. Generally, the data rate is inversely proportional with the distance, and this is due the decrease in SNR in the reflected path, which is inversely proportional to the distance from the RIS. The realistic RIS phase shift model is adopted, and the data rate performance gap is evident when $\beta _{\min }$ is changed from the ideal case which is $\beta _{\min }=1$ to the actual case which is $\beta _{\min }=0.2$. We notice that the achievable data rate in the positional phase profile exhibits slightly better performance than the directional phase profile, although the difference between the two is negligible. Nevertheless, both of them outperform the random phase case and this is expected since the random phase has only an aperture gain with no passive beamforming gain enhancement.

Figure 11 illustrates the achievable data rates for various phase configurations in relation to the number of RIS elements at SNR of 25 dB and a distance of 12 meters from the RIS. Our investigation focuses on assessing the impact of the amplitude-phase shift model for different $\beta _{\min }$ values, specifically $\beta _{\min } \in \{0.2,1\}$, on the achievable data rate. In general, we observe a decline in the achievable rate when $\beta _{\min }<1$. Firstly, it is evident that the performance of the random phase shift approach at the RIS remains unaffected by the number of elements. This result aligns with expectations, given that random phase shifts only contribute to aperture gain without any passive beamforming gain. Secondly, it is notable that both positional and directional phase configurations exhibit superior performance compared to the scheme employing random phase shifts. As the number of elements increases, we observe a consistent rise in achievable rates. This trend is logical, as the passive beamforming performance steadily improves with the increasing number of elements.

## Conclusion

We addressed the task of localizing the user in a 3D space using a RIS-based lens with a practical phase-shift model. By leveraging the curved nature of the wavefront, it is possible to estimate the location of the user by utilizing multiple phase configurations of the RIS. Fisher information analysis offers valuable insights into the design of these phase configurations taking into consideration the actual RIS phase shift model. Additionally, we have introduced a low-complexity 3D localization algorithm that simplifies the problem by decoupling the main 3D problem into 3 1D problems using the angular expansion approach. The advantage of this work shows the realistic results when using the practical phase-dependent amplitude model. The literature is saturated with RIS-related communication and localization works with overoptimistic results and far-field assumptions so, we tried in our work to prove that considering the lossless (ideal phase shift model) is not accurate in measuring the performance of localization (such as PEB and RMSE) and communication (such as achievable data rate). There are various potential research directions that can be explored further. One such direction involves mutual coupling and electromagnetic interference. When the RIS sections on the substrate material are in close proximity, effectively maintaining separation poses a challenge. This proximity gives rise to mutual coupling, wherein the impedance of one element becomes interconnected with the impedances of its neighboring elements. Consequently, the frequency response will vary depending on the configuration of the adjacent elements. Several researchers overlook the electromagnetic interference inevitably present in any environment, choosing instead to concentrate solely on the signals produced by the system. Electromagnetic interference can stem from various sources, including natural occurrences, intentional activities, or unintentional factors like man-made devices and background radiation. Generally, every unregulated wireless transmission produces electromagnetic interference. The energy of electromagnetic interference waves impacting the RIS in the space in front of it is directly proportional to its surface area. When the RIS emits the absorbed electromagnetic interference energy again, it can reach the intended receiver, leading to a degradation in the SNR of wireless networks that are unaware of such interference effects. The decline in SNR is attributed to the fact that these wireless networks or systems are primarily designed to counteract only the thermal noise generated by the receiver, neglecting the impact of external electromagnetic interference. Considering mutual coupling and electromagnetic interference could provide additional accurate information and insights for proper positioning and communication. These areas offer promising opportunities for advancing localization and communication techniques and enhancing their performance in practical scenarios.

## Data Availability

The data generated or analysed during this study are available from the corresponding author on reasonable request.
